# Quantitative Shear-Wave Elastography of the Liver in Preterm Neonates with Intra-Uterine Growth Restriction

**DOI:** 10.1371/journal.pone.0143220

**Published:** 2015-11-18

**Authors:** Marianne Alison, Valérie Biran, Anca Tanase, Matthieu Bendavid, Marie Blouet, Charlie Demené, Guy Sebag, Mickael Tanter, Olivier Baud

**Affiliations:** 1 Department of Pediatric Radiology, Robert Debré Children University Hospital and Denis Diderot Paris University, APHP, 75019 Paris, France; 2 PremUP foundation, 75014 Paris, France; 3 Neonatal Intensive Care Unit and INSERM U1141, Robert Debré Children University Hospital and Denis Diderot Paris University, APHP, 75019 Paris, France; 4 Institut Langevin, CNRS UMR 7587, INSERM U979, ESPCI ParisTech, 75005 Paris, France; Rensselaer Polytechnic Institute, UNITED STATES

## Abstract

The feasibility and reproducibility of liver stiffness measurements using Supersonic Shear-wave Imaging (SSI) in preterm neonate have not been reported. Our aim was to determine if liver stiffness differs between intra-uterine growth restriction (IUGR) and appropriate for gestational age (AGA) preterm infants with/without cholestasis. We measured liver stiffness (in kPa) in 45 AGA and 18 IUGR preterm infants, and assessed reproducibility in 26 preterms using Intraclass Correlation Coefficients (ICC) and Bland-Altman tests. Liver stiffness values were compared between AGA and IUGR with and without cholestasis and correlated with birth weight. Measurements showed high reproducibility (ICC = 0.94–0.98 for intra-operator, 0.86 for inter-operator) with good agreement (95% limits: -1.24 to 1.24 kPa). During the first postnatal week, liver stiffness was higher in IUGR (7.50 ±1.53 kPa) than in AGA infants (5.11 ±0.80 kPa, p<0.001). After day 8, liver stiffness remained unchanged in AGA but increased progressively in IUGR infants (15.57 ±6.49 kPa after day 21). Liver stiffness was higher in IUGR neonates with cholestasis (19.35 ± 9.80 kPa) than without cholestasis (7.72 ± 1.27 kPa, p<0.001). In conclusion, quantitative liver SSI in preterms is feasible and reproducible. IUGR preterms who will develop cholestasis present high liver stiffness even at birth, before biological cholestasis occurs.

## Introduction

Intra-uterine growth restriction (IUGR) is a frequent and major challenge in pregnancy care [1]. It is associated with stillbirth, neonatal mortality and perinatal morbidity leading to potential long-term handicap and diseases, even in adulthood [2–4].

In most cases, IUGR occurs due to utero-placental insufficiency, which can lead to chronic fetal hypoxia, damaging developing organs including the liver. The fetal liver is mainly supplied with oxygenated blood by the umbilical vein [5]. In IUGR, the hepatic artery and portal vein partially compensate for reduced umbilical flow, but liver oxygenation is reduced [6, 7], with an increased risk of hypoxic injury leading to liver disease.

Due to immaturity of mechanisms involved in bile formation, the newborn is more susceptible to develop cholestasis. Transient neonatal cholestasis is the most frequent liver dysfunction observed in IUGR neonates. It can be exacerbated by prolonged parenteral nutrition and leads to significant post-natal co-morbidities [8–11]. It is characterized by early-onset occurrence, absence of a known cause of neonatal cholestasis, normalization of clinical and biochemical parameters during follow-up, and an history of some neonatal injurious event (asphyxia, sepsis, total parenteral nutrition, etc.). B-mode ultrasound is usually normal [12].

We know that several perinatal factors impact the liver development in case of IUGR: abnormal prenatal growth and metabolism, postnatal inflammation, prolonged parenteral nutrition. To date, no reliable early markers are available to predict the occurrence of cholestasis and related complications in IUGR preterm infants. We hypothesized that liver stiffness, a good predictor of liver disease in adult [13], could serve as an early diagnostic marker of neonatal liver insults, and would be increased in preterm IUGR newborns with transient cholestasis.

Several quantitative sono-elastography techniques have been developed to assess the mechanical properties of liver tissue and to stage fibrosis in adults and children [14]. Transient elastography was the first and most widely used technique [15, 16] and acoustic radiation force impulse (ARFI) the second [17–20]. Supersonic Shear-wave Imaging (SSI) is the third and provides a real-time, quantitative 2D image of stiffness [21–23]. SSI combines the remote generation of a shear vibration through acoustic radiation [24] and its transient imaging using ultrafast ultrasonic techniques [25]. It allows the generation of a quantitative elasticity color map of biological tissues in a single acquisition session (<20 ms) with a corresponding conventional real-time B-mode image. Clinical potential of SSI has been demonstrated in chronic liver disease in adults [13, 26, 27] but has not yet been evaluated in neonates.

The aim of this study was therefore to assess:

1. the feasibility and reproducibility of liver stiffness measurements using SSI in preterm neonates,
2. whether liver stiffness is different in IUGR preterms and preterms with a birth weight appropriate for gestational age (AGA),
3. whether liver stiffness differs between IUGR and AGA preterms with and without cholestasis.

## Materials and Methods

### Patients

This prospective observational study was approved by our Institutional Review Board (IRB) and local ethics committee of Robert Debré children's hospital. All parents provided informed oral consent. IRB approved oral consent because elastography images of the liver were obtained simultaneously to B-mode image acquisition, considered as a standard of care. This consent has been reported in the clinical record.

Between January 2013 and September 2013, 63 preterm neonates born before 36 weeks of gestation were enrolled in the study. Patients with surgically-treated malformations, chromosomal aberrations or antenatal viral infections were excluded. Patients were recruited and split into two groups according to their birth weight:

- 18 preterm neonates born with severe IUGR (IUGR group) with a birth weight below the 3^rd^ percentile according to customized French curves (AUDIPOG: Association des Utilisateurs de Dossiers Informatisés en Pédiatrie, Obstétrique et Gynécologie) [28],
- 45 preterm neonates with a birth weight appropriate for gestational age (AGA group).

Patients were further divided according to biological analysis: IUGR with cholestasis, IUGR without cholestasis, AGA with cholestasis, AGA without cholestasis. Cholestasis was defined as serum conjugated-bilirubin concentrations above 30 mmol/L (1.75 mg/dL) within the first 3 postnatal weeks.

For secondary analyses, population was split into two gestational age groups (24−31^6/7^ weeks of gestation for very preterm neoanates and 32−36^6/7^ weeks of gestation for moderately preterm neonates).

### Liver imaging

Liver imaging was performed in neonatal intensive care unit in each patient at least once, before postnatal day 8, between postnatal days 8 and 21 or after postnatal day 21. Eighteen patients were imaged at all time points (8 IUGR and 10 AGA neonates). When a patient underwent several measurements during the same period, the mean value of all time points was used for analysis.

Two pediatric radiologists with 5 and 10 years of experience in pediatric imaging and 6 to 12 months of experience with SSI technique performed hepatic B-mode ultrasound, doppler analysis and SSI using the Aixplorer® ultrasound scanner (Supersonic Imagine, Aix-en-Provence, France) with a linear L10-2 probe (256 elements, 6 MHz central frequency).

Liver B-mode analysis included gallbladder, biliary tract and liver morphology (size, contours, echogenicity and homogeneity) to rule out differential diagnoses of hepatic cholestasis such as choledochal cysts or lithiasis.

Longitudinal diameters of the liver and spleen were compared to normal ranges in preterm infants [29].

Liver stiffness was measured (in kPa) on quantitative elasticity maps by the 2 experienced pediatric radiologist blinded to clinical conditions of the neonates.

SSI technique has been described in detail in previous publications [21, 25]. Briefly, a shear vibration is generated in the tissue by focused ultrasound beams that induce mechanical tissue displacements of a few tens of μm, and imaged at very high frame rates (up to 20,000 frames/s) to calculate shear wave propagation speed (v) and the local elastic modulus (Young modulus, E) based on the following equation: E ≈ 3μ = 3ρv^2^_T_, where μ is the shear modulus and ρ is the density. The resulting elasticity maps are displayed on a color scale ranging from 0 to 240 kPa. Spatial resolution is 1 mm^2^ [25].

Liver stiffness measurements were performed using a subcostal approach, under free breathing, in a patient in supine position. The probe was positioned over different segments of the liver using conventional real-time B-mode imaging, and SSI sequence measurement launched to obtain real-time 2D elasticity maps.

**Fig 1:**
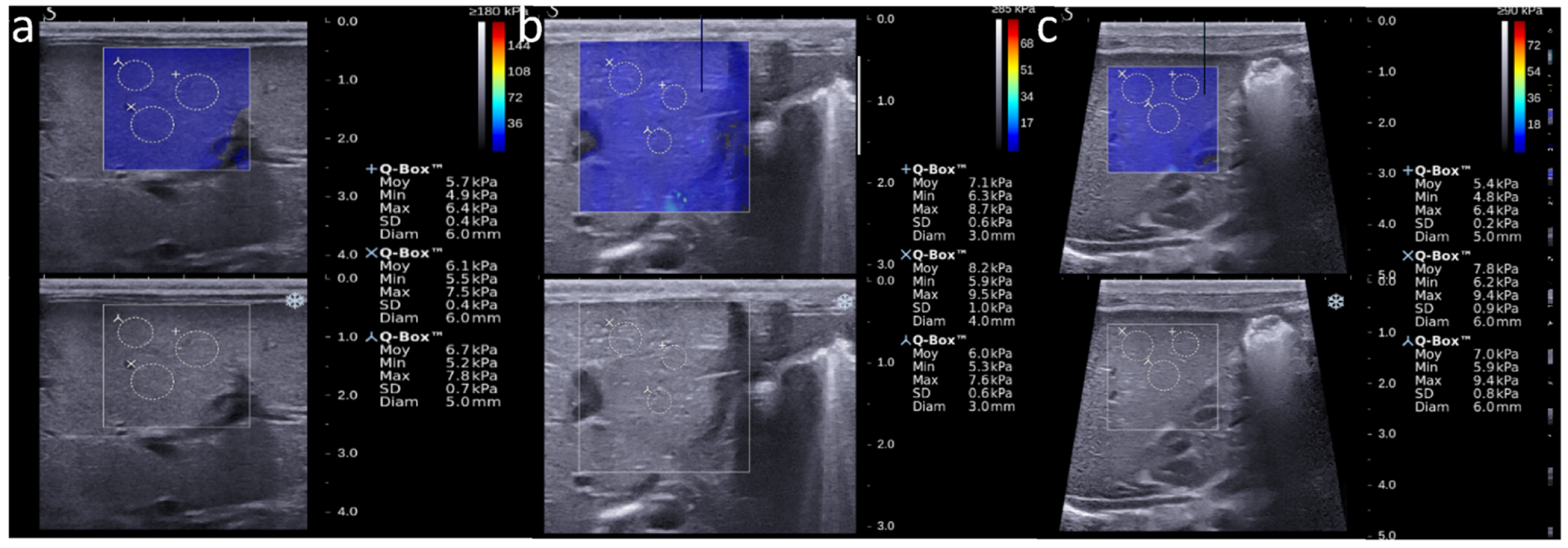
Quantitative measurement of liver stiffness in different liver areas. A: Subcostal view in the axial plane demonstrating the left portal branch and ROIs in segment IV, with overlying elasticity color map. B: Subcostal view in the axial plane demonstrating the portal bifurcation and ROIs in segment III, with overlying elasticity color map. C: Subcostal right sagittal view demonstrating the kidney and ROIs in segment VI, with overlying elasticity color map.

Two separate acquisitions were obtained from three different liver segments according to the nomenclature of Couinaud and Bismuth (axial view of segment III including the left portal branch, axial view of segment IVb demonstrating the portal bifurcation and sagittal view of segment VI demonstrating the right kidney), to obtain 6 elasticity maps (Fig 1). Three round regions of interest (ROIs) (0.5–1cm^2^) were manually positioned by the operator on each elasticity map, avoiding subcapsular areas and vessels. Mean stiffness value (± standard deviation) was calculated for each ROI, and the 18 ROI values were averaged for each patient.

### Statistical analysis

Overall statistical analyses were performed using Medcalc software (MedCalc Statistical Software v12.7.7; Ostend, Belgium; http://www.medcalc.org; 2013) and GraphPad Prism software (GraphPad Prism, v5.0, San Diego, California, USA). The significance level was set at p < 0.05.

#### Reproducibility and repeatability of stiffness measurements (SSI)

Intra- and inter-observer reproducibility of measurements was evaluated in the first 26 patients. For intra-observer reproducibility, acquisition and measurements were repeated twice by the same observer during the same imaging session. For inter-observer reproducibility, the 2 pediatric radiologists with 6 to 12 months of experience in SSI performed acquisitions and measurements on the same day. The mean stiffness value for each patient was calculated for each operator, and reproducibility of measurements calculated using a Bland-Altman test [30] and analysis–of-variance intra-class correlation coefficient (ICC) [31, 32]. Reproducibility was classified as excellent (ICC ≥ 0.75), fair-to-good (ICC = 0.40–0.75) or poor (ICC ≤ 0.40) [33]. For Bland–Altman tests, the bias (mean difference) and 95% limits of agreement (confidence interval) are given.

Repeatability was defined as the variation in repeated measurements in the same subject under identical conditions [34], and was quantified separately for each operator as the average coefficient of variation (standard deviation/mean) of repeated measurements.

#### Comparison of stiffness values between IUGR and AGA infants with and without cholestasis

Mean liver stiffness values in AGA and IUGR neonates according to the presence or absence of cholestasis were compared using one-way ANOVA with Dunnett’s correction. For comparisons of repeated measures over time, two-way ANOVA (based on a mixed model) was calculated with time point at assessment and group of patients (IUGR and AGA infants with and without cholestasis) as factors, followed by a Newman-Keuls post-hoc test.

#### Correlation of liver stiffness values during the first week of life with birth weigh

Liver stiffness measured within the first week of life was correlated to birth weight (expressed in percentiles according to gestational age) for preterm infants born before or after 32 weeks of gestation. Pearson's correlation coefficients have been calculated to assess whether the severity of fetal growth restriction or prematurity were risk factors for elevated liver stiffness.

## Results

### Characteristics of the population studied

The main characteristics of the population studied are summarized in Table 1. Despite a slightly higher gestational age at birth in the IUGR group, differences between the 2 groups did not reach statistical significance.

**Table 1:** Characteristics of the populations studied.

	AGA (N = 45)	IUGR (N = 18)
Gestational age at birth (weeks, mean [1–3 quartiles])	28.5 [26–31]	30 [28–33]
Gestational age < 32 weeks of gestation	34 (76%)	11 (61%)
Multiple gestation	12 (27%)	9 (50%)
Male	24 (53%)	14 (78%)
Birth weight (g, mean [interquartile range])	1120 [890–1400]	920 [725–1193]
Death	2 (4%)	2 (11%)
Respiratory distress syndrome	31 (69%)	8 (44%)
Chronic lung disease at 36 weeks	8/43 (19%)	6/16 (38%)
Secondary sepsis	16 (36%)	6 (33%)
Patent ductus arteriosus	13 (29%)	2 (11%)
Severe brain lesions	4 (9%)	2 (11%)
Parenteral nutrition duration (days, mean [interquartile range])	36 [18–51]	31 [16–120]
Biological cholestasis	5 (11%)	7 (39%)

AGA: appropriate for gestational age; IUGR: intra-uterine growth retardation

Biological cholestasis (defined as serum conjugated-bilirubin concentrations above 30 mmol/L / 1.75 mg/dL) occurred significantly more often in the IUGR than in the AGA group (7/18 [39%] vs. 5/45 [11%], p = 0.01), although the mean duration of parenteral nutrition was not statistically different (Table 1).

### 2D liver imaging and Doppler

In IUGR neonates, B-mode liver imaging and Doppler were initially normal in all patients, with no biliary tract abnormalities, abnormal hepatic structure or signs of portal hypertension. In one IUGR patient hepatomegaly was detected from postnatal day 46, and splenomegaly with 2 small hepatic nodules from postnatal day 60. Ascites was found after postnatal day 21 in 4 out of 18 IUGR patients. In all AGA neonates, liver and abdominal imaging and Doppler analyses were normal.

### Liver stiffness (SSI)

#### Feasibility

Liver stiffness could be measured in all patients and in all liver areas, even in patients with ascites. Acquisitions were free of the excessive image motion or poor beam coupling seen in B-mode imaging. No issues with penetration have been observed in preterm neonates.

#### Reproducibility and repeatability of liver stiffness measurements in neonates

In the first 26 patients, used to assess reproducibility, liver stiffness values ranged from 4.0 to 7.7 kPa. Mean stiffness was not statistically different between operators (5.60 ± 0.84 vs. 5.60 ± 0.97, p = 0.99).

**Fig 2:**
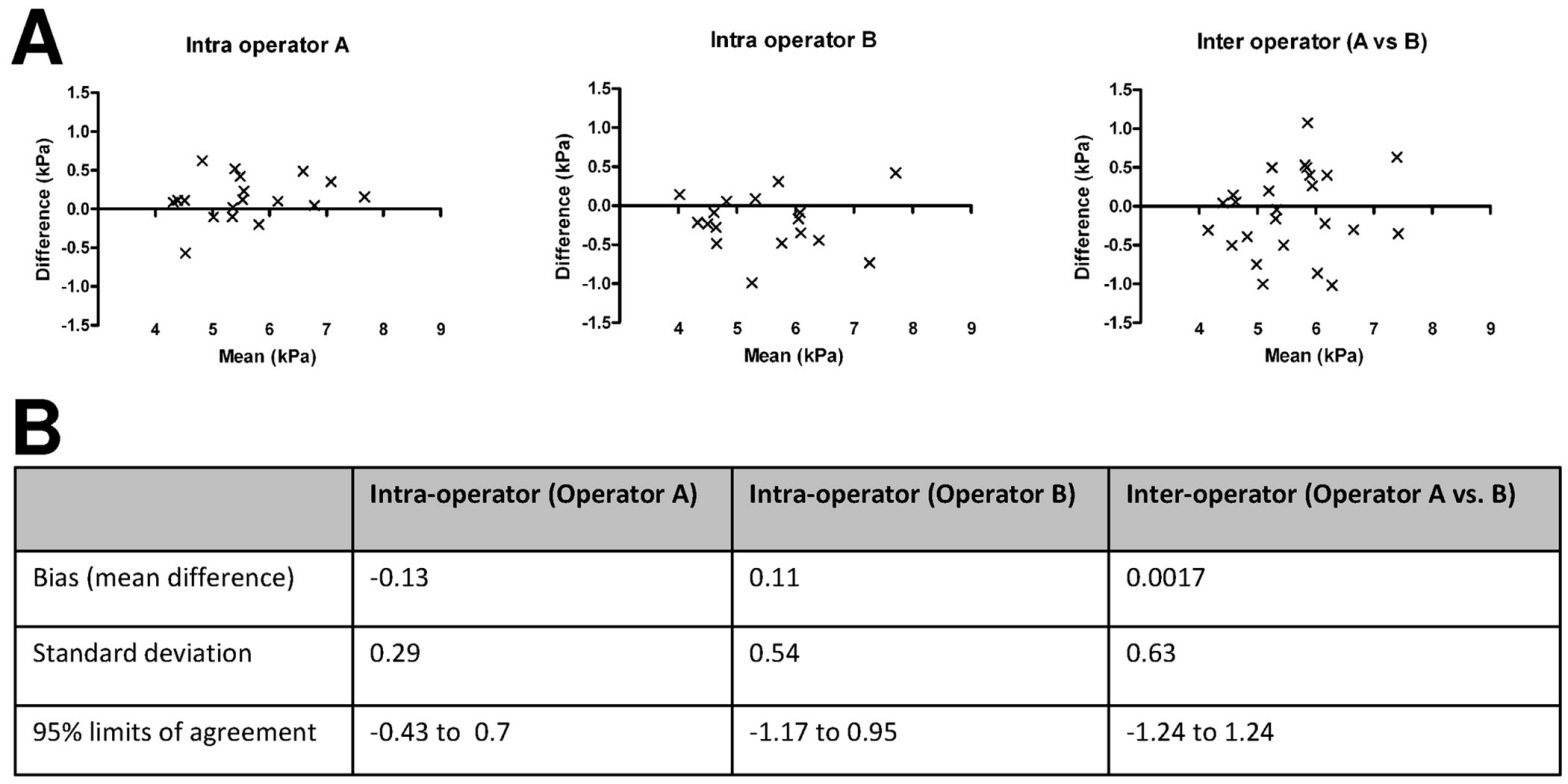
Reproducibility and repeatability of liver stiffness measurements in neonates. A: Bland-Altman plot for intra-observer (operators A and B) and inter-observer (A versus B) reproducibility of liver stiffness measurements (kPa). B: Intra- and inter-observer reproducibility of measurements.

Intra-operator reproducibility was excellent for both operator A (ICC = 0.96) and operator B (ICC = 0.88). The within-session repeatability of liver stiffness measurements was 6.7% for operator A and 4.6% for operator B. Inter-operator reproducibility was also excellent (ICC = 0.76). Bland-Altman plot are given in Fig 2A. Mean bias and limits of agreement are given in Fig 2B.

#### Comparison of liver stiffness values between IUGR and AGA

**Fig 3:**
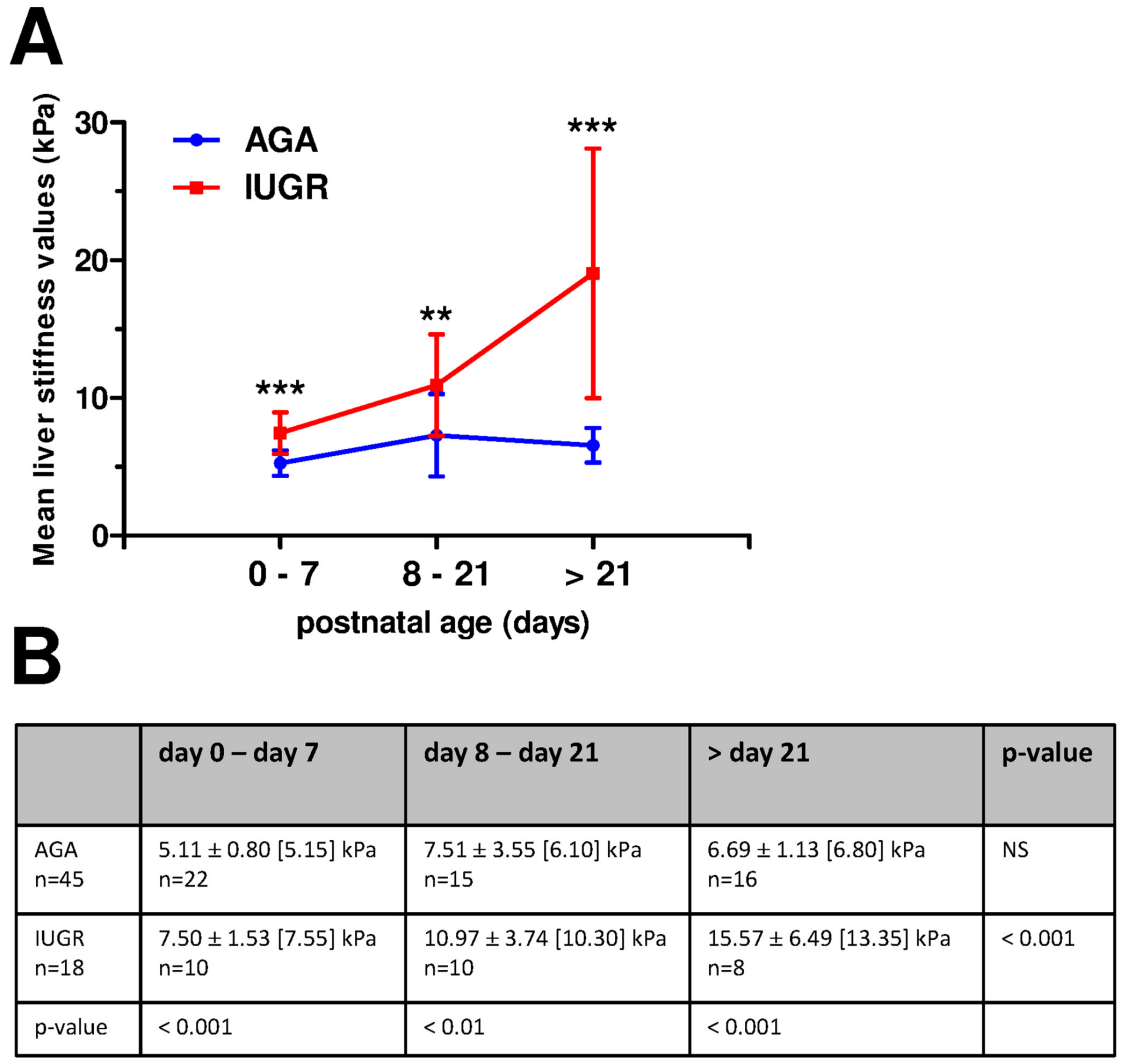
Comparisons of liver stiffness values (kPa) at three postnatal periods in AGA and IUGR neonates. A: Dot plots represent means and standard deviations (SD). **:p<0.01, ***:p<0.001, using two-way analysis of variance performed with time points at assessment and group (AGA or IUGR) as factors, followed by a Newman-Keuls post hoc test. B: Mean (± SD) [median] liver stiffness (kPa) for AGA and IUGR neonates according to postnatal age. When several measurements were performed in the same patient during a given period, the mean stiffness value was used for analysis. Repeated measures two-way analysis of variance (mixed model) was performed with time points at assessment and group (AGA or IUGR) as factors, followed by a Newman-Keuls post hoc test.

During the first week of life, liver stiffness was significantly higher for IUGR (n = 10; mean ± standard deviation: 7.50 ± 1.53 kPa) than for AGA infants (n = 22; 5.11 ± 0.80 kPa, p<0.001), whereas biological markers of liver disease were normal. After day 8, liver stiffness in AGA patients remained stable whereas liver stiffness in IUGR neonates continued to increase (Fig 3).

#### Liver stiffness during the first week of life, severity of IUGR and prematurity

**Fig 4:**
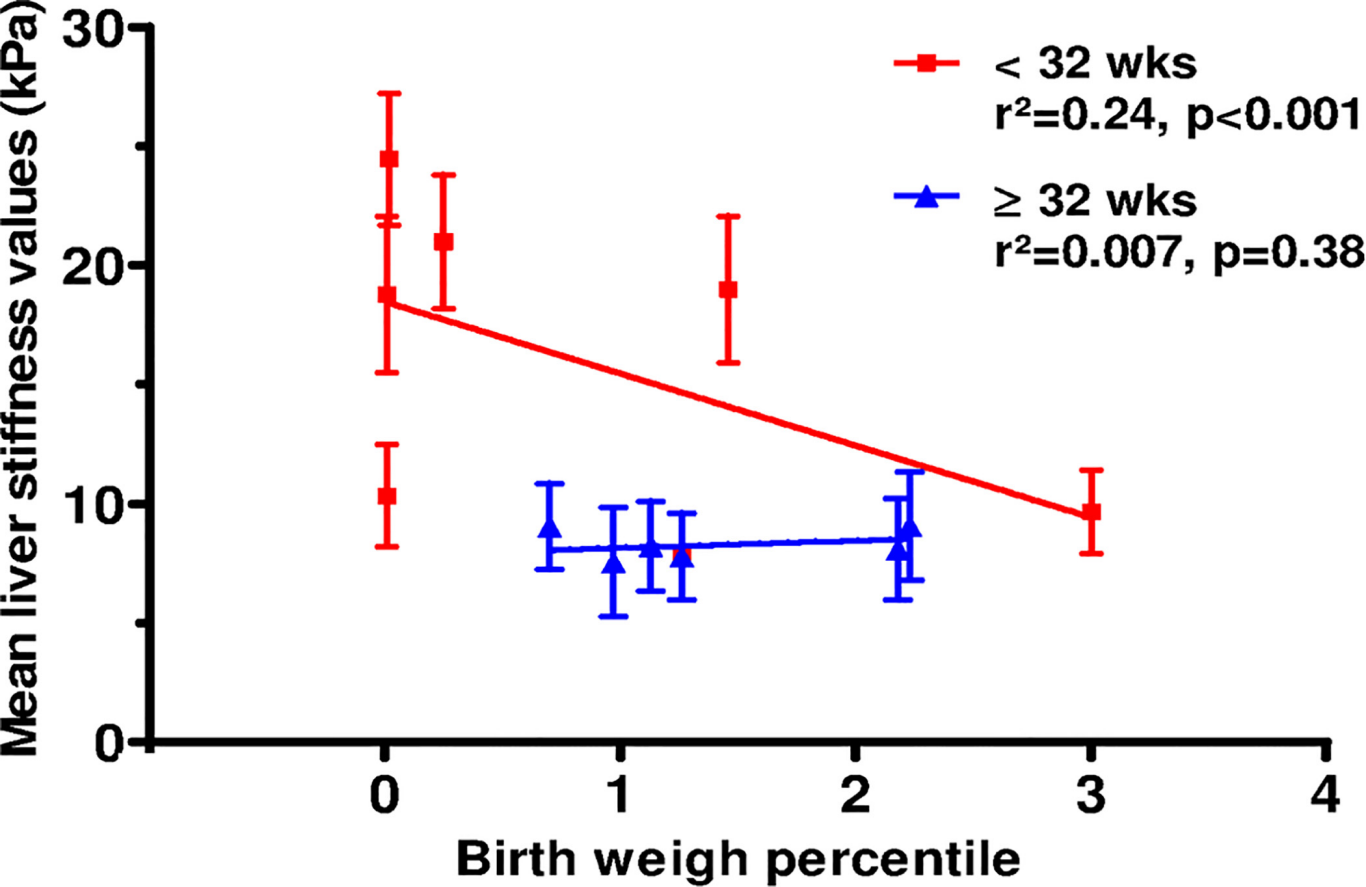
Mean liver stiffness values according to birth weight percentile for IUGR neonates born before and after 32 weeks of gestation. Linear regression indicates r^2^ = 0.244 (p<0.001) for IUGR neonates born before 32 weeks of gestation and r^2^ = 0.007 (p = 0.38) for IUGR neonates born after 32 weeks of gestation.

Liver stiffness measured within the first week of life was negatively correlated with birth weight percentile for IUGR infants born very preterm (p<0.001, Fig 4). In contrast, the severity of IUGR did not appear to be significantly associated with increased liver stiffness in infants with a gestational age above 32 weeks.

#### Liver stiffness and cholestasis

**Fig 5:**
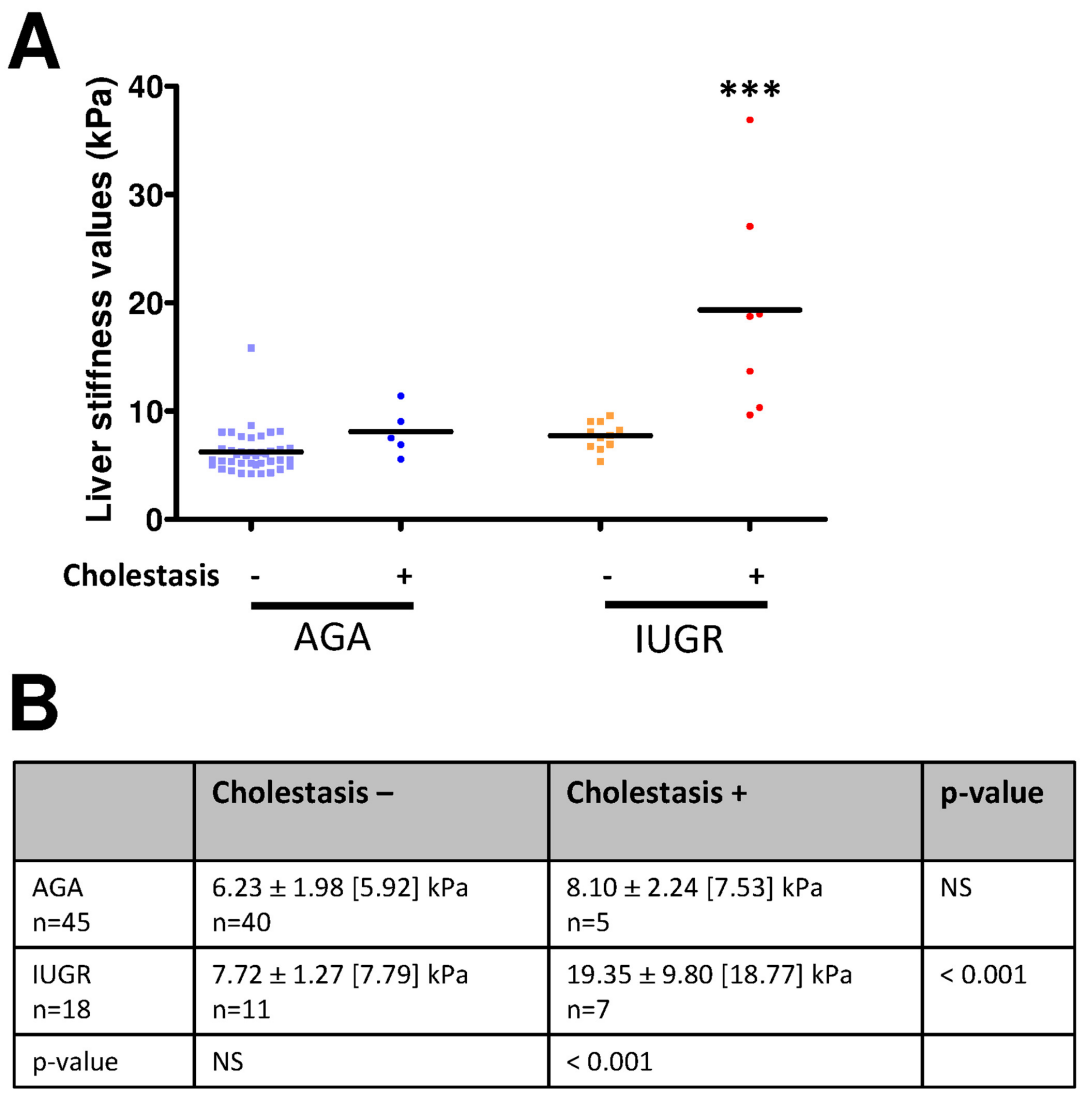
Liver stiffness, IUGR and cholestasis. A: Liver stiffness values (kPa) in AGA and IUGR neonates with (+) and without (-) cholestasis. When several measurements were carried out in the same patient at different time points, the highest value of all time points was used for analysis. ***: p<0.001 using one-way ANOVA with Dunnett’s correction. B: Mean (± SD) [median] liver stiffness in AGA and IUGR neonates according to the presence or absence of cholestasis. For each patient, the highest value measured was used for analysis. Indeed, elevated liver stiffness associated with cholestasis appears to be a transient process over time. Analysis comparisons between the groups were performed using one-way ANOVA with Dunnett’s correction.

Average liver stiffness value was significantly higher in IUGR preterms with cholestasis (19.35 ± 9.80 kPa) than without cholestasis (7.72 ± 1.27 kPa, p<0.001) during hospitalization stay. In contrast, it was not significantly higher in AGA preterms with cholestasis (8.10 ± 2.24 kPa) than without cholestasis (6.23 ± 1.98 kPa) (Fig 5). For the 4 out of 18 surviving IUGR patients who presented with ascites related to severe cholestasis after postnatal day 21, the mean liver stiffness value was found very high (22.2 ± 11.8 kPa).

#### Liver stiffness and clinical outcome

**Fig 6:**
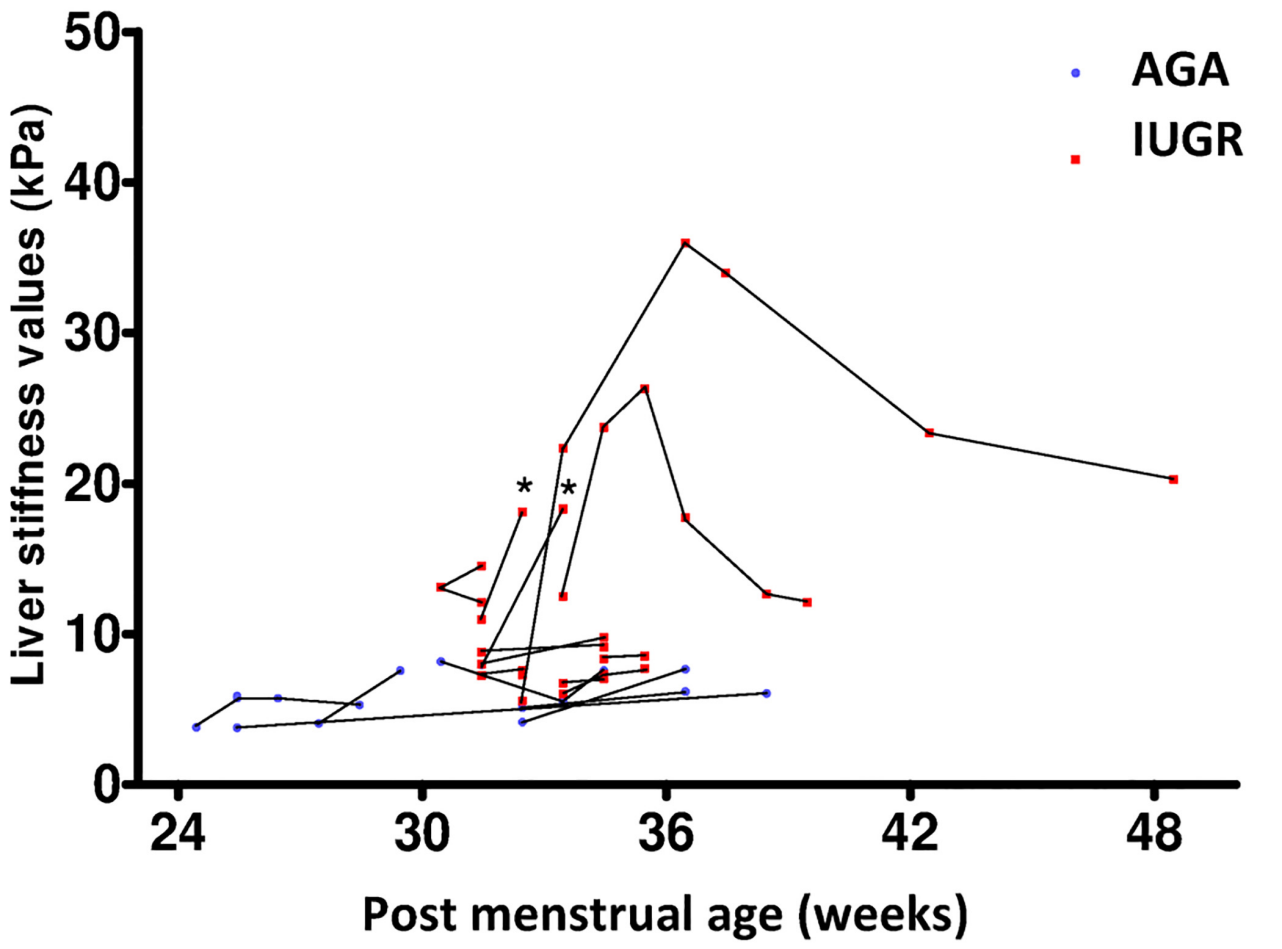
Follow-up of liver stiffness values for neonates imaged at all time points. Asterisks indicate neonatal deaths.

Two IUGR neonates with elevated liver stiffness died during follow-up (Fig 6). This neonates are indicated by asterisk in the figure. For the other IUGR patients with cholestasis (n = 5), biological recovery occurred before liver stiffness decreased.

In the 2 cases with the highest liver stiffness, stiffness remained high after the normalization of biological markers of cholestasis, with the persistence of adverse clinical events related to liver dysfunction (ascites, infections…) for 6 and 9 weeks respectively. The patient with the highest liver stiffness presented changes in liver morphology (hepatomegaly and hepatic nodules) and signs of portal hypertension (splenomegaly and ascites) on follow-up.

## Discussion

Our study demonstrates that quantitative stiffness measurement with SSI is both feasible and reproducible in preterm neonates. Moreover, this technique provides further informations associated to B-mode ultrasound in detecting liver changes in this population, and shows that liver stiffness is higher in IUGR compared to AGA preterms, as well as being higher in IUGR neonates with cholestasis compared to IUGR without cholestasis.

Besides other ultrasound-based techniques used to quantify tissue stiffness, SSI combines both quantification and real-time B-mode imaging, strongly improving its ease-of-use and the detection of potential artifacts (unstable in time) in elastographic images.

We demonstrated here that SSI provides many medical interests in preterm neonates including better prediction of neonates with IUGR prone to develop cholestasis within the first week of postnatal life and better assessment of liver disease course despite biological recovery of transient cholestasis in IUGR.

No report on normal liver values in neonates explored using SSI has been reported to date. The liver stiffness values of AGA newborns in our study (ranging from 5.11 ± 0.80 to 7.51 ± 3.55 kPa) were within the same range as those measured previously in normal adults using the same technique: from 4.89 ± 0.33 [35] to 6.00 ± 1.40 kPa [36]. Liver stiffness was increased in IUGR neonates with biological cholestasis (19.35 ± 9.80 kPa), probably corresponding to liver microstructural changes. ARFI and Fibroscan technique have been also reported higher elastography values in neonatal liver diseases including biliary atresia [16, 18, 20]. In contrast to most of these chronic liver diseases with fibrosis, increased elastography observed in neonatal transient cholestasis is unlikely related to fibrosis as it was found reversible. In IUGR patients, liver stiffness was negatively correlated with birth weight percentile for preterms born before 32 weeks of gestation, suggesting that IUGR and prematurity are additive risk factors for increased liver stiffness.

Transient neonatal cholestasis is a complication of parenteral nutrition, with multiple risk factors. Neonates are more susceptible to parenteral-nutrition-associated cholestasis because of their physiological immaturity, with a reported incidence as high as 40–60% [37]. Both IUGR and prematurity are associated with the need for protracted parenteral nutrition, and impaired fetal growth is one of the main conditions that predisposes preterms to developing postnatal cholestasis [8–11]. Indeed, IUGR causes hepatocellular dysfunction, leading to a decreased capacity to metabolize proteins during the first postnatal weeks of life and to altered hepatic fatty acid metabolism [9, 38].

Relationship between transient cholestasis and transient increased liver stiffness in our study remains to be elucidated. We speculate that it could be due to reversible liver microstructural changes related to IUGR but we have no histological evidence to confirm this hypothesis. Few studies have reported postnatal histopathological changes in transient neonatal cholestasis. Jacquemin et al. found moderate portal and lobular fibrosis, multinucleated giant hepatocytes, and hematopoietic foci with improvement or normalization on follow-up liver biopsy specimens [12]. IUGR neonates receiving parenteral nutrition and developing liver failure demonstrate extensive portal and sinusoidal fibrosis [8, 39].

Increased liver stiffness is related to fibrosis in most chronic liver diseases. In neonates and infants, increased liver stiffness has been reported in biliary atresia with liver fibrosis [16, 18, 20]. Besides fibrosis, hepatic inflammation, congestion or cholestasis have also been previously reported to increase liver stiffness [40–42].

In our cohort, the dispersion of liver stiffness values increases with higher values, probably due to an inhomogeneous distribution of the liver damage. This has already been demonstrated in adults with different liver diseases such as fibrosis, inflammation, congestion or cholestasis [43].

Several potential limitations of our study should be taken into account:

1. this is a proof-of-concept study that investigated a relatively small number of subjects in a single center,
2. liver stiffness changes could occur *in utero* due to fetal growth restriction, but also postnatally during prolonged parenteral nutrition in sick neonates, two separate issues that could confound one another,
3. we defined IUGR using the most recent customized French curves for male and female neonates separately; however optimal weight was not calculated using a fetal weight standard individually adjusted for physiological pregnancy variables, as recommended by Gardosi et al. [44],
4. we did not compare right to left liver lobe measurements despite reports showing that stiffness values in the left lobe of the liver were higher and inhomogeneous using transient elastography and ARFI techniques [45],
5. finally, another limitation is the lack of long term follow-up of our cohorts.

## Conclusion

In conclusion, liver stiffness measurement using SSI is feasible in preterm neonates and showed high intra-operator and inter-operator reproducibility, with good agreement. This technique could be a useful tool for the early discrimination and follow-up of IUGR preterm neonates at higher risk of developing transient neonatal cholestasis. Detecting these patients can potentially improve their neonatal care.

## Abbreviations

SSI: shear-wave imaging
IUGR: intra-uterine growth restriction
AGA: appropriate for gestational age
ICC: intraclass correlation coefficients
